# An Intelligent Multimode Clustering Mechanism Using Driving Pattern Recognition in Cognitive Internet of Vehicles

**DOI:** 10.3390/s21227588

**Published:** 2021-11-15

**Authors:** Huigang Chang, Nianwen Ning

**Affiliations:** 1 State Key Laboratory of Networking and Switching Technology, Beijing University of Posts and Telecommunications, Beijing 100876, China; 2 School of Artificial Intelligence, Henan University, Zhengzhou 450046, China; nnw@henu.edu.cn

**Keywords:** Cognitive Internet of Vehicles, artificial intelligence, autonomous driving, genetic algorithm, clustering mechanism

## Abstract

Connected autonomous vehicles can leverage communication and artificial intelligence technologies to effectively overcome the perceived limitations of individuals and enhance driving safety and stability. However, due to the high dynamics of the vehicular network and frequent interruptions and handovers, it is still challenging to provide stable communication connections between vehicles, which is likely to cause disasters. To address this issue, in this paper, we propose an intelligent clustering mechanism based on driving patterns in heterogeneous Cognitive Internet of Vehicles (CIoVs). In the proposed approach, we analyze the driving mode containing multiple feature parameters to accurately capture the driving characteristics. To ensure the accuracy of pattern recognition, a genetic algorithm-based neural network pattern recognition algorithm is proposed to support the reliable clustering of connected autonomous vehicles. The cognitive engines recognize the driving modes to group vehicles with a similar driving mode into a relatively stable cluster. In addition, we formulate the stability and survival time of clusters and analyze the communication performance of the clustering mechanism. Simulation results show that the proposed mechanism improves the reliable communication throughput and average cluster lifetime by approximately 14.4% and 11.5% respectively compared to the state-of-the-art approaches.

## 1. Introduction

The Intelligent Transportation System (ITS) benefits from communication and intelligence technologies to enhance traffic management capabilities and improve vehicle driving efficiency. Autonomous driving (AD) as an important component of ITS has received widespread attention due to its ability to avoid traffic jams, reduce the probability of traffic accidents, and free human hands, which will produce significant economic and social benefits. At present, the Connected Autonomous Vehicles (CAVs) employ vehicular communication technology to exchange environmental and perception information to obtain non-line-of-sight (NLOS) traffic information to strengthen its own perception capabilities [1]. In addition, the analysis of a large amount of sensory data in network space and physical space can effectively assist CAVs in AD decision making [2,3,4,5]. However, the traditional Internet of Vehicles (IoV) merely provides communication services and lacks efficient cognition of physical space and network space, thus preventing intelligent vehicle mobility management and collaboration to better support AD services [6,7].

Recently, the Cognitive Internet of Vehicle (CIoVs) has emerged as an innovative paradigm for bringing intelligence and cognitive capabilities to IoV, which can provide smarter, reliable, and proactive services in AD scenarios [8]. CIoVs emphasizes the use of cognitive methods to facilitate intelligent collaboration in physical and network space to ensure traffic safety and quality of communication services. The cognitive engine (CE) can assist decision making and guide the movement of CAVs. In addition, the powerful CAVs also provide a wealth of onboard communication, sensing, and computing resources for AD scenarios. For instance, CAVs can share safety warnings, perceptual information, and control instructions to ensure the safety of AD. Furthermore, orchestrating CAVs into different clusters through active collaboration can support more efficient and safer swarm intelligence services and reduce fuel consumption [9,10,11].

Although CIoVs can make AD services and applications become more intelligent and efficient, there are still changes in vehicle mobility management and reliable connectivity of communications. Firstly, dynamic changes in the vehicular network topology may cause unreliable and unstable communication connectivity. Since CIoVs contain a large number of connected devices, many applications are processed locally or at the edge, which requires a more flexible network architecture. In addition, as safety and non-safety services require different network performance, singular communication technology cannot meet the quality of communication services. Furthermore, the mobility of CAVs leads to frequent interruptions and handovers in communication due to changes in access points. It is difficult to guarantee a continuous and consistent communication service, which is essential for the security of network-assisted AD [12,13,14].

To address these issues, in this paper, we propose an intelligent clustering mechanism based on driving behaviors in heterogeneous CIoVs, which introduces CE to recognize AD mode and perform clustering to better support network-assisted AD scenarios. We consider a connected autonomous driving scenario where the vehicles are CAVs equipped with communication devices to investigate the communication performance gains obtained through intelligent clustering among vehicles. Currently, fully connected AD is not yet completely accessible due to policy, regulation, and technical constraints. However, the powerful sensing capabilities of CAVs and Artificial Intelligence (AI) technologies can already support both non-connected and connected vehicles coexistence scenario. With the further development of technology and improvement of policies and regulations, the penetration rate of CAVs will further increase and eventually achieve a fully driverless scenario.

In the proposed mechanism, the CE analyzes driving behavior to classify CAVs into different clusters based on the similarity metrics of AD modes. In addition, a leadership assessment indicator is designed to select the CAVs with the highest leadership priority as the cluster head (CH). Different communication technologies are utilized inside and outside the cluster, which has dedicated short-range communication (DSRC) and 5G-V2I technologies [15,16]. It enables high connectivity and wide area coverage to meet the communication requirements of both safety and non-safety services [17,18]. The proposed mechanism allows for more stable clusters through CAVs collaboration, reduces communication interruptions and switching caused by random movements, and ensures communication connectivity and reliability. The main contributions of this paper are summarized as follows:

- We design a heterogeneous CIoVs network architecture and propose an intelligent clustering mechanism using AD pattern recognition, which brings CAVs with the same or a similar driving mode together to form a stable cluster to enhance the connectivity of the communication service.
- We establish an AD mode model considering multiple driving parameter factors to perform AD pattern recognition. Then, the stability and survival time metrics of clusters are formulated and analyze the communication performance of the proposed clustering mechanism.
- To ensure the effectiveness of clustering, we propose a genetic algorithm (GA)-based neural network (GANN) AD pattern recognition algorithm to perform accurate AD mode recognition for reliable clustering.
- The simulation results are discussed and analyzed to evaluate the effectiveness of the proposed clustering mechanism compared with existing schemes in terms of AD pattern recognition accuracy, communication throughput, and average cluster lifetime.

The rest of the paper is organized as follows. Related works are introduced in Section 2. Section 3 presents the network architecture, clustering mechanism, and driving behavior modeling. Section 4 describes the GANN algorithm for AD pattern recognition in detail. Section 5 analyzes the performance indicators of the proposed clustering mechanism. Simulation results and discussions are carried out in Section 6. Finally, we summarize this paper in Section 7.

## 2. Related Work

As important components of smart city and ITS, IoV and AD have attracted extensive interest from both industry and academia. In this section, we discuss related work from the perspective of the vehicular network architecture, the network clustering and switching mechanism in IoV, and vehicle clustering and the driving behavior modeling approach that are closely related to this paper. The comparative study of our work with existing work is listed in Table 1.

### 2.1. IoV Network Architecture

Lin et al. [19] proposed a software-defined networking (SDN) enabled vehicular network architecture which divided the networks into three layers to improve network management capacity. In [20], the authors proposed a cognitive radio (CR)-based architecture for in-vehicle networks to employ CR technology to alleviate spectrum resource shortages in the presence of highly dynamic typologies and time-varying spectrum utilization. They used reinforcement learning methods to design optimal data transmission scheduling schemes and make full use of V2V and V2I communication resources. To deal with the problem of a lack of communication between vehicles, the authors in [21] proposed a heterogeneous V2V communication architecture and investigated a relaying method for the coexistence of DSRC and LTE-V2V technologies. In [22], the authors investigated the performance of the heterogeneous IoV architecture with multiple communication modes, which included V2V-only and hybrid situations. In [23], the authors surveyed a heterogeneous vehicular network architecture, which utilized multiple wireless communication technologies. Through analyzing and comparing the performance indicators required by safety services and non-safety services, they pointed out that a heterogeneous vehicular network combined with a cellular network and DSRC would be a potential solution to meet the communication requirements of ITS. These articles have put forward some communication optimization schemes from the aspect of vehicular network architecture and data transmission, which include multiple communication technologies and modes. However, the dynamic changes in network topology and communication interruptions and handovers caused by the random mobility of vehicles still challenge the reliability and scalability of IoV.

### 2.2. Network Clustering and Switching in IoV

Qi et al. [24] proposed a traffic differentiated clustering routing mechanism for vehicular data collection to reduce cellular bandwidth cost in a heterogeneous network, which was compose of DSRC and cellular vehicle-to-everything. To achieve high reliability, low latency, and wide-area coverage communication, the authors in [25] proposed an adaptive clustering method, which utilized DSRC and the cellular network to provide intra-cluster V2V communication and V2I communication outside the cluster, respectively. In [26], the authors proposed a section-based cluster mechanism, which clustered vehicles based on road sections and selected the vehicle closest to the cluster center as the CH, ignoring the driving stability of vehicles. In [27], the authors proposed a dynamic cluster adjustment mechanism to improve the scalability of the vehicular network, where CH discovered malicious cluster member (CM) nodes and adjusted cluster size according to the available spectrum to maximize resource utilization. The authors in [28] proposed a recommendation and switching mechanism in heterogeneous IoV to alleviate the load on the cellular network and improve network performance. In [29], the authors proposed an online reinforcement learning approach, which enabled switching between different heterogeneous vehicular networks by observing the patterns of data traffic in the spatial-temporal dimension. These studies investigated the communication switching problem caused by the dynamic network topology of the IoV from the perspective of clustering of heterogeneous networks. However, they neglected the impact of driving behavior and patterns of vehicles in heterogeneous vehicular networks on the communication performance, which is crucial for the stability of vehicle clusters and the reliability of communication.

### 2.3. Vehicle Clustering and Driving Behavior Modeling

Wang et al. [30] proposed a classification method for driving encounter scenarios using connected vehicle trajectories. A generic unsupervised learning framework including a feature representation layer and a clustering layer was designed to cluster driving encounter scenes based on multi-vehicle GPS trajectories. In [31], the authors employed machine learning methods to cluster the vehicle trajectories from a network perspective, which constructed IoV based on k-nearest neighbor. In [32], the authors adopted density-based spatial clustering of application with a noise algorithm to analyze travel patterns in a city and evaluate the similarity between trajectories. In [33], the authors proposed a clustering approach based on history and current driving information for ITS, which utilized the social relationship to perform a clustering algorithm. In addition, a vehicle route selection algorithm based on game evolution was proposed to control the traffic flow. In [34], the authors employed an internal reward function-based driving model to simulate human decision-making mechanisms and proposed a structural hypothesis on human driving behavior focused on discrete potential driving intentions. Tan et al. [35] proposed a new approach to unify the modeling of driving behavior in different scenarios, which combined behavioral theories and field theory. Liu et al. [36] investigated a driving behavior scoring model based on entropy weighting and hierarchical analysis, which analyzed driving behavior data by establishing a driving behavior identification algorithm. Shahverdy et al. [37] proposed a convolutional neural network based method to recognize five types of driving styles according to vehicle signals instead of monitoring driver visual features. These articles focused on vehicle clustering in physical space and behavioral analysis in a single traffic scenario; however, they lacked joint optimization and analysis of communication performance in a connected autonomous driving scenario.

Although much work has been done on IoV, traditional IoV ignores the driving characteristics and intelligence of CAVs, which leads to poor vehicle communication connectivity that cannot serve AD well. In network-assisted AD scenarios, CIoVs can better support AD scenarios by introducing the CE for cognition and collaboration in physical and cyberspace. Specifically, the cognition and clustering of the driving pattern can effectively improve the stability of the cluster, reduce frequent network switching and interruptions, and provide stable and reliable communication. In addition, effective collaboration between CAVs can greatly enhance the safety and efficiency of AD. However, it is very complex and challenging to solve the above problems.

In this paper, we propose an AD pattern recognition algorithm and design an intelligent clustering mechanism based on different AD patterns to provide stable and reliable AD communication services.

## 3. Network Architecture and Clustering Mechanism

In this section, we describe the proposed architecture and the intelligent clustering mechanism based on AD pattern recognition in detail. First, we present the proposed system architecture of the heterogeneous CIoV, the components that are included, and their functions in detail. We then describe the process of modelling AD patterns and build a training model which serves as the basis for pattern recognition. Finally, we detail the pattern recognition based clustering and CH selection mechanism.

### 3.1. CIoVs Architecture for AD

**Figure 1:**
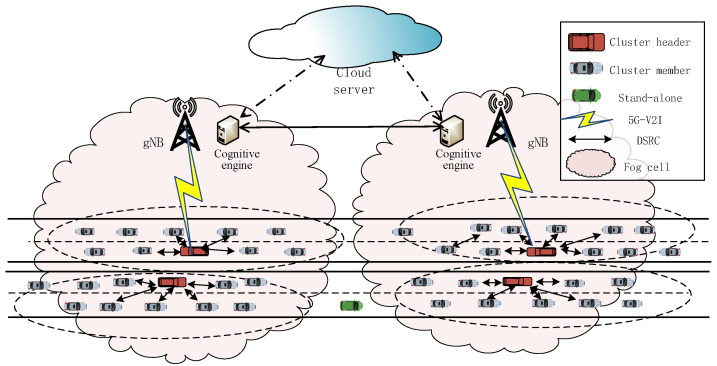
Heterogeneous CIoVs architecture.

The proposed reliability-enhanced hierarchical CIoVs architecture is shown in Figure 1. The architecture mainly contained multiple CAVs with different AD modes, a 5G base station gNB, CE, and a cloud server (CS). Within the fog cell, the gNB collects Floating Car Data (FCD) within its coverage area. The CE processes the collected FCD to perform AD pattern recognition and clustering. Then, the CAVs are divided into different clusters according to AD modes. Among them, CAVs with the same or similar AD mode can form a cluster to maintain a relatively stable state. When the driving characteristics of CAVs are different from the proposed AD modes or a new driving mode appears, it can be switched to the same mode as the cluster member vehicles. Specifically, the constituents and functions are as follows.

(1) CAVs: CAVs are classified into CHs, CMs, and standalone (SA) vehicles, where SA vehicles refer to vehicles that are not clustered. Specifically, DSRC is an effective V2V communication technology, which is used to support the communication between vehicles in the cluster to provide realtime safety services. The SA CAVs communicate directly with the base station.
(2) gNB: The gNB is a centralized communication infrastructure that is responsible for communication with CHs and SA vehicles. For example, outside the cluster, the CH connects with the gNB through 5G-V2I technology, which can use the existing communication infrastructure to access the CS to obtain wider communication coverage and internet services.
(3) CE: The CE can perform AD mode recognition and control the switching of different modes based on context and location information to ensure cluster stability and driving efficiency. For example, when a new mode appears, it can be switched according to the prevailing situation and driving needs.
(4) CS: The cloud server can obtain delay-insensitive information from the gNB and provide global traffic information and services. We adopt two main communication technologies to complete different communication services, namely DSRC and 5G-V2I.

The CAVs set *V* within a fog cell is expressed as $V=\left\{V_{1},V_{2},V_{3},\ldots ,V_{N}\right\}$, where N is the total number of vehicles. In the fog cell, CAVs generate a large amount of FCD and networking data. Processing these data to obtain valuable information can better promote ITS and network-assisted AD. These CAVs are divided into multiple clusters $C=\left\{C_{1},C_{2},\ldots ,C_{L}\right\}$ according to the excavated driving mode information. The vehicles in the fog cell have three different AD modes, and the set of driving modes can be expressed as $V\_ID=\left\{M_{1},M_{2},M_{3}\right\}$, where $M_{1}$ represents conservative driving mode, $M_{2}$ represents moderate driving mode, and $M_{3}$ represents aggressive driving mode. Therefore, the category attribute of the vehicle can be expressed as $V_{i\_v}\in V\_ID$. The modeling of the AD mode will be introduced in the next subsection.

The gNB collects FCD and passes it to CE and the CE uses machine learning (ML) algorithms for data analysis to obtain the AD mode attributes of CAVs. The ML algorithm was used in this paper, which has strong data fitting and function mapping capabilities, and feature classification can be performed efficiently and reasonably [38]. Then, the CAVs with the same or similar driving patterns are gathered together to form a cluster through control instructions. Finally, each cluster selects the most reliable vehicle as the CH to connect with the gNB according to the stability of the AD mode, location, and communication status. This clustering mechanism can reduce the burden on the base station network and provide reliable communication [39]. Clustering is performed based on the result of AD pattern recognition, and  we model the AD mode in the next subsection.

### 3.2. AD Mode Modeling

The AD modes of CAVs should be human-like and need to be adapted to the prevailing situation in order to achieve a comfortable and safe experience [40,41]. Based on the characteristics of driving in real urban traffic conditions, we classified the driving modes into three modes: aggressive, moderate, and conservative.These contain the main driving characteristics and can meet the needs of most driving scenarios. The CAVs with aggressive mode will have a relatively high-speed distribution compared to the CAVs with the other two modes and will overtake the CAVs of the other two modes. Conservative mode is the opposite of aggressive mode, i.e., lower average speed, very steady state of motion, and a tendency to follow. Finally, the moderate mode is between them.

Considering the driving behavior of the vehicle changes dynamically over time, we used VISSIM simulation software to collect the driving data of vehicles in different driving modes over time, which is presented in Section 6.1. Finally, through statistical analysis and preprocessing of these data, we obtained multidimensional data information closely related to the driving modes, i.e., feature vectors. The reflection in speed obeys truncated Gaussian distribution, the speed and overtaking frequency are mainly affected by the independent variables of acceleration and acceleration time. Therefore, based on the above analysis, several eigenvalues most relevant to the AD mode were selected as training data, including normalized velocity, acceleration time, overtaking frequency, etc. Through statistical analysis of the driving data of vehicles $v_{i}$ in different modes, the preprocessed statistical feature value was used as the training feature tensor. The preprocessed statistical feature values were employed as training feature vectors. The feature matrix can be expressed as

(1) $$F={\left[\begin{array}{ccccccc} A_{v_{1}} & B_{v_{1}} & C_{v_{1}} & D_{v_{1}} & E_{v_{1}} & F_{v_{1}} & T_{1}\\ A_{v_{2}} & B_{v_{2}} & C_{v_{2}} & D_{v_{2}} & E_{v_{2}} & F_{v_{2}} & T_{2}\\ \vdots & \vdots & \vdots & \vdots & \vdots & \vdots & \vdots \\ A_{v_{i}} & B_{v_{i}} & C_{v_{i}} & D_{v_{i}} & E_{v_{i}} & F_{v_{i}} & T_{3} \end{array}\right]}^{T},$$

where $v_{i}\in V$ and the meaning of each letter parameter can be expressed as follows: $A_{v_{i}}$ represents the mean of the velocity distribution of the CAV $v_{i}$, $B_{v_{i}}$ represents the variance of the velocity distribution, $C_{v_{i}}$ represents the acceleration mean, $D_{v_{i}}$ represents the acceleration variance, $E_{v_{i}}$ represents the acceleration time, $F_{v_{i}}$ represents the overtaking frequency. T represents the vector of the output label. The GANN algorithm for AD pattern recognition is proposed in the next section, which utilizes these feature tensors as the training sets to identify the similarity of AD modes for intelligent clustering of CAVs and the clustering mechanism is introduced in the following subsection.

### 3.3. Clustering Mechanism

The model divided the AD mode into three categories, and the driving mode category label of each CAVs and its probability value in the category were obtained through the proposed AD pattern recognition algorithm. In the same category, the larger the probability value, the more stable the driving mode. It can be expressed as

(2) $$V\_ID=\left\{\begin{array}{c} M_{1}=1\\ M_{2}=2\\ M_{3}=3 \end{array},\right.$$

(3) $$P\left(v_{i\_v}\right)=\left[P\left(v_{i\_v}\in V\_ID\right)\right]=GANN\;\left(v_{i\_v}\right).$$

Among them, V_ID represents the vehicle driving mode category and $P(v_{i\_v})$ represents the output category vector of the proposed driving pattern recognition algorithm GANN, i_v=1,2,…,N. It is a classification probability value, which satisfies $P\left(v_{i\_v}\in M_{1}\right)+P\left(v_{i\_v}\in M_{2}\right)+P\left(v_{i\_v}\in M_{3}\right)=1$. The vector $V_{i\_v}=\left(V\_ID,\max P\left(v_{i\_v}\right)\right)$ represents the driving mode category attribute of CAV i_v, where $\max P(v_{i\_v})$ is the probability value of vehicle i_v under the category V_ID, which reflects the stability of the driving mode. Therefore, the driving mode category indicator of the vehicle can be expressed as

(4) $$S_{v_{i\_v}}=V\_ID+\max P\left(v_{i\_v}\right),$$

where $S_{v_{i\_v}}$ represents the quantitative value of the category attribute of the vehicle driving mode. The larger the value of $S_{v_{i\_v}}$, the higher the stability of the vehicle in the category. If CAV i_v belongs to $M_{1}$ category; $S_{v_{i\_v}}\in \left(1,2\right)$; if CAV i_v belongs to $M_{2}$ category, $S_{v_{i\_v}}\in \left(2,3\right)$; if CAV i_v belongs to the $M_{3}$ category, $S_{v_{i\_v}}\in \left(3,4\right)$.Then, according to the recognized vehicle mode, the vehicles with the same category are grouped in the same cluster. At the same time, in the same category, the larger the value of $S_{v_{i\_v}}$, the higher the priority for being selected as the CH. Based on the proposed clustering mechanism, in the next subsection we will introduce the cluster header selection method.

### 3.4. Cluster Header Selection

CAVs with the same AD mode will not have frequent overtaking and switching behaviors because the CAVs in the cluster have similar speed distributions and have already met their own driving needs. In addition, to improve cluster stability and flexibility, the CAV can be switched to different AD modes based on traffic and road conditions. Appropriate CH can improve the reliability of communication inside and outside the cluster and avoid frequent switching due to CH changes. When clustering is performed, CAVs with the same V_ID value are grouped together to form a cluster based on position. The leader priority can be defined based on stability, channel quality, and distance from the centroid and it can be expressed as

(5) $$L_{v_{i\_v}}\left(t\right)=c_{1}\left|10(\max P\left(v_{i\_v}\right)+\rho \left(t\right))\right|+c_{2}\left|D_{norm}\right|\;t=1,2,\ldots ,T,$$

where ρ(t) represents the channel quality index, $D_{norm}$ is the normalized distance of each vehicle from the cluster center, and $c_{1}$ and $c_{2}$ are the weights of each variable, $c_{1}+c_{2}=1$. When the value of $\max P(v_{i\_v})$ is larger, the driving state of the vehicle in this mode is more stable. It can be seen that when the vehicle with the largest $L_{v_{i\_v}}\left(t\right)$ is selected as the CH, the subsequent ones will be used as candidate CH.

## 4. GANN for AD Pattern Recognition

In this section, we establish an AD pattern recognition model optimized by GA that takes full advantage of NN and GA.

### 4.1. AD Pattern Recognition

#### 4.1.1. NN Model for AD Pattern Recognition

According to the driving model established above, the NN architecture for AD pattern recognition was established as follows:

(a) Number of layers in the NN: The NN consists of an input layer, an output layer, and multiple hidden layers. Theoretical studies have shown that a three-layer network with one hidden layer, which has sufficient neurons can achieve arbitrary nonlinear mapping [42]. We adopted a three-layer structure for pattern recognition.
(b) Number of neurons in the input layer: The input to the NN is the dimension of the feature vector associated with the driving mode. Here we chose six parameters that were closely correlated to our proposed AD mode as the input vector, i.e., the input layer had six neurons. Through the statistical analysis, the six parameters, respectively, correspond to [A,B,C,D,E,F].
(c) Number of neurons in the output layer: The matrix of the target output column vectors corresponding to each category is $\left[T_{1};T_{2};T_{3}\right]$. Specially, $T_{1}$ stands for aggressive mode, which means that the vehicle accelerates frequently and travels at a high speed. $T_{3}$ stands for conservative mode, which means that the vehicle tends to actively brake to maintain a longer following distance and travels at a lower speed. $T_{2}$ stands for moderate mode and refers to the transition zone between the above two modes, which usually maintains a steady speed with little fluctuation. The NN target output column vector $T_{1}$, $T_{2}$, $T_{3}$ are $\left[1,0,0\right]$, $\left[0,1,0\right]$, $\left[0,0,1\right]$, respectively. Therefore, the number of neurons in the output layer is three.

**Figure 2:**
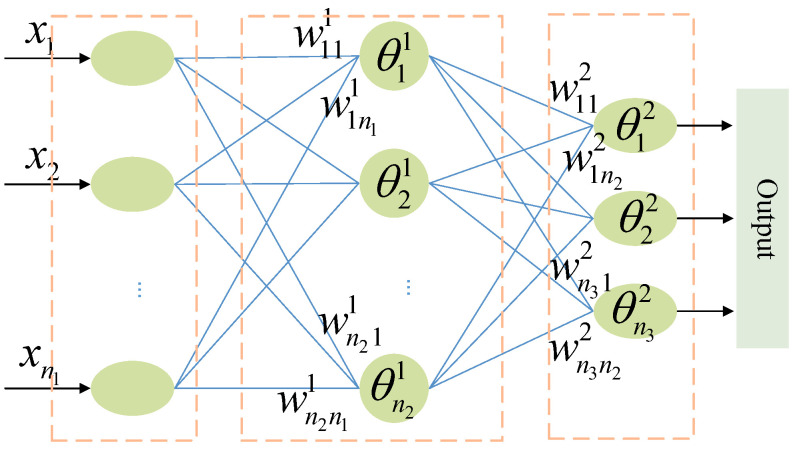
Neural network topology.

The driving pattern recognition model is a fully connected feedforward NN as shown in Figure 2. It works according to the principle that the number of neurons in the hidden layer is larger than that of the input layer and proportional. Considering the tradeoff between benefit and efficiency, the parameters and variables of the proposed GANN for AD pattern recognition are shown in Table 2.

#### 4.1.2. Cost Function

According to the proposed network structure, the NN weights and thresholds to be optimized can be expressed as a matrix as follows

(6) $$W=[w^{1},\theta^{1},w^{2},\theta^{2}],$$

where, *W* is the total parameter to be optimized; $w^{1}$ is the weight between the input layer neurons and the hidden layer neurons; $\theta^{1}$ is the threshold of the hidden layer neurons; $w^{2}$ is the weight between the hidden layer and the output layer neurons; and $\theta^{2}$ is threshold of the output layer neurons.

The error of the actual output value and the target value of the network is taken as the objective function. The error between the expected value and the output value can be continuously reduced by using the gradient descent method. The error cost function of NN can be expressed as

(7) $$E=\frac{1}{2m}\sum_{k=1}^{n_{3}}{\left(Y_{k}-Z_{k}\right)}^{2},$$

where *m* represents the number of training samples, $Y_{k}$ is the target value of the training sample, and $Z_{k}$ is the actual output value of the NN. In the process of network forward propagation, the weights and thresholds were first initialized. Using vectorization operation to calculate the output of each layer, the output of the hidden layer neurons and the output of the output layer neurons are, respectively, expressed as

(8) $$O_{j}=f\left(w^{1}x_{i}-\theta_{j}^{1}\right)=f\left(net_{j}\right)\;i=1,\ldots ,n_{1},$$

(9) $$Z_{k}=g\left(w^{2}O_{j}-\theta_{k}^{2}\right)=g\left(net_{k}\right)\;k=1,\ldots ,n_{3}.$$

where $j=1,\ldots ,n_{2}$ and f(⋅) and g(⋅) are the transfer functions of the hidden layer and the output layer, respectively. After two layers of forwarding transmission, the actual output value of the network can be obtained, and the network error can be obtained by substituting the above formula. The NN error function can be expressed as

(10) $$E=\frac{1}{2m}{\sum_{k=1}^{n_{3}}\left\{Y_{k}-g\left[\sum_{j=1}^{n_{2}}w_{kj}^{2}f\left(\sum_{i=1}^{n_{1}}w_{ji}^{1}x_{i}-\theta_{j}^{1}\right)-\theta_{k}^{2}\right]\right\}}^{2}.$$

This shows that the NN error is a function of the parameters with respect to the *W*. Therefore, the pattern recognition error can be reduced by continuously adjusting the *W* of the NN.

#### 4.1.3. Parameter Update

Using the backpropagation algorithm, the *W* updates along the direction of the gradient descent of the error function until the error accuracy requirement is met or the maximum number of iterations is reached. The update process of weights and thresholds can be expressed as follows

(11) $$\left\{\begin{array}{c} w\left(t+1\right)=w\left(t\right)-Y\frac{\partial E}{\partial w}\\ \theta \left(t+1\right)=\theta \left(t\right)+Y\frac{\partial E}{\partial \theta } \end{array}\right.,$$

where Y is the learning rate, i.e., the adjustment step size of the weights and thresholds. The partial derivative of the model error function can be calculated using the chain derivation rule. However, the initial *W* has a large impact on the performance of the NN model, which will result in unstable classification results. In order to obtain the global optimal solution, the initial *W* needs to be optimized.

### 4.2. GANN Algorithm for AD Pattern Recognition

The optimization process includes the NN training phase and GA optimization phase. In this subsection, we use the GA algorithm to optimize the initial *W*. First, the *W* to be optimized was encoded as the chromosome of individuals, and the NN error norm was used to construct the fitness function. Then, we calculated the fitness value of individuals in the population, and used the operations of selection, crossover, and mutation to generate the next-generation and find the optimal chromosome. The specific steps of the GANN algorithm are as follows:

(1) Population  initialization: The population contains *M* individuals, each of which represents all the parameters to be optimized. Each individual can be coded as
  (12) $$Ind=\left\{w^{1},\theta^{1},w^{2},\theta^{2}\right\},$$
  where Ind represents an individual in the population and *M* is the number of individuals included in the population during initialization. That is, all weights and thresholds are encoded as a chromosome of an individual, which is a row vector. Then a population of size *M* can be expressed as
  (13) $$X=\left[Ind_{1};Ind_{2};\ldots ;Ind_{M}\right],$$
  where *X* represents the initial population of the GA. Len represents the chromosome length of each individual. During the initial coding of the individuals, each parameter is coded in a standard binary code. That is, all the parameters to be optimized for each individual are encoded as a 10−bit binary number, so the length of the chromosome of each individual can be represented as $L_{en}=10\times \left(252+42+126+3\right)\;=4230$.
(2) Fitness function determination: The fitness function is established based on the objective function. According to the principle of the survival of the fittest by GA, the smaller the error of the objective function, the greater the fitness value. Based on the above analysis, the fitness function can be expressed as
  (14) $$Fit\left(X\right)=\frac{2}{E},$$
  where *X* represents an individual in the population and *E* represents the test sample error produced by the individual.
(3) Selection operation: The GA will preferentially select individuals with higher fitness values for inheritance, and the superior genes of the previous generation will be preserved. The probability that a parent individual is selected is
  (15) $$P\left(X_{Ind}\right)=\frac{Fit_{m}}{\sum_{m=1}^{M}Fit_{m}}.$$
  The probability of selection is constructed according to the individual fitness value, i.e., individuals with high fitness values are more likely to pass on the genes to the next generation.
(4) Crossover operatio: Using a single point crossover method, each parental individual produces a progeny individual through chromosome crossover. Assuming that the parent individuals are $C_{u}\left(gen\right)$ and $C_{v}\left(gen\right)$, respectively, the progeny individuals generated after the crossover are
  (16) $$C_{v}\left(gen+1\right)=\left\{\begin{array}{c} C_{v}\left(gen,q\right)\;\;q<r_{c}\\ C_{u}\left(gen,q\right)\;\;q\geq r_{c} \end{array}\right.,$$
  (17) $$C_{u}\left(gen+1\right)=\left\{\begin{array}{c} C_{u}\left(gen,q\right)\;\;q<r_{c}\\ C_{v}\left(gen,q\right)\;\;q\geq r_{c} \end{array}\right..$$
  That is, the parent forms new offspring by exchanging chromosomes, where $r_{c}\in \left[1,L_{en}\right]$ is the crossover coding lengths of chromosomes.
(5) Mutation operation: In order to maintain the global search characteristics of GA and avoid the convergence to the local optimum quickly, the individuals must maintain a certain probability of variation. Mutation produces the number of variant genes with a certain probability, and randomly selects the gene for mutation. If the selected gene is coded as 1, it becomes 0; otherwise, it becomes 1. Variations maintain population diversity to prevent immature convergence.
(6) Termination condition: We set *G* as the maximum number of genetic generations of the GA. The optimization algorithm terminates when the GA reaches the maximum genetic generation or minimum target error. Then, the individual that has the smallest error in the genetic evolution process is the optimal solution. The output of the final training result is the optimal solution.

### 4.3. GANN for AD Pattern Recognition Algorithm

Algorithm 1 presents the process of GANN for pattern recognition, which overcomes the defect that NN easily falls into the local optimal during the learning process.
**Algorithm 1** GANN for AD pattern recognition**Input:** Initial population *X*, Training samples *F*, Initialization parameters.**Output:** Optimal network parameters *Ind*, minimum error *E*.1:NN structure determination.2:Initialize model parameters $\left\{n_{1},n_{2},n_{3},G,M,\cdots ,X\right\}$.3:$X=\left[Ind_{1};Ind_{2};\cdots ;Ind_{M}\right]$.4:Convert *X* to decimal, calculate the objective function value *E*.5:gen=0.6:**while***gen* ≤ *G* do7: Training of NN;8: Calculate Fit(X);9: Calculate P(X), then selection, crossover, and mutation operation get $X^{\prime }\left(Ind\right)$;10: Convert $X^{\prime }\left(Ind\right)$ to decimal, compute $E\left(X^{\prime }\right)$;11: $X\left(gen+1\right)=X\leftarrow X^{\prime }$;12: Convert X(gen+1)→decimal;13: gen=gen+1;14: Update trace← best error *E*, optimal *X*;15:**end while**

The main idea was to use the test error obtained through NN training as the optimization objective function of GA. We will introduce the NN training part in the next section. The NN model is a multi-classification problem. The *Softmax* function has good multi-classification properties, it is a probability selection mechanism. At the output layer of NN, we used *Softmax* as the transfer function. It can be expressed as

(18) $$p\left(z_{k}\right)=\frac{e^{z_{k}}}{\sum_{k=1}^{n_{3}}e^{z_{k}}}.$$

The sum of the probabilities of the output of the three categories is 1, and the class with the highest probability can be output as the mode category to which the CAV belongs. A larger output value indicates a higher accuracy of the classification, where the NN training process for AD to obtain the error of each iteration is the objective function of GA optimization. The training of NN is to minimize network output errors in each training process by updating the weights and thresholds.

## 5. Performance Analysis

In this section, we introduce the stability and lifetime of the cluster and analyze the communication performance of the proposed clustering mechanism.

### 5.1. Cluster Stability and Lifetime Analysis

Considering the CAVs in motion is a time-varying process, each period *T* is divided into several equal time slots τ. Each time slot is used to collect the beacon information of the vehicle, which can be obtained from the electronic control unit (ECU). Therefore, the vehicle position information $V_{i\_v}\left(t\right)=\left[x_{i\_v},y_{i\_v}\right]$ and other driving parameters can be used for cluster stability calculation and driving mode classification. For example, the vehicle speed and the distance between vehicles can be effectively calculated based on the location information of the vehicle at a certain moment.

The relative distance between $CH_{j\_v}$ and $CM_{i\_v}$ can be obtained through the Euclidean formula using position information. It can be characterized as

(19) $$D_{i\_v,j\_v}\left(t\right)=\sqrt{{\left(x_{i\_v}-x_{j\_v}\right)}^{2}+{\left(y_{i\_v}-y_{j\_v}\right)}^{2}}.$$

The average relative distance between $CM_{i\_v}$ and $CH_{j\_v}$ in a cluster can be expressed as $D_{v}\left(t\right)=\frac{1}{\left|c\right|}\sum_{c}(V_{i\_v}\left(t\right)-V_{j\_v}\left(t\right))$. Therefore, the sequence of the average distance variable between $CM_{i\_v}$ and $CH_{j\_v}$ changing with time is $\left\{D_{v}\left(1\right),D_{v}\left(2\right),\cdots ,D_{v}\left(t\right)\right\}$, which can be obtained using the Euclidean distance. We can obtain $\Delta D_{V}\left(t\right)=D_{v}\left(t+1\right)-D_{v}\left(t\right)$ by performing the difference operation on the average relative distance $D_{v}\left(t\right)$, then the stability index of the cluster can be derived by calculating the variance of the difference variable $\Delta D_{V}\left(t\right)$. The stability index of the cluster can be expressed as

(20) $$S_{ta}=Var\left(\Delta D_{V}\left(t\right)\right).$$

When the CM of the cluster changes, the average distance between CH and CM will change, even the CAV will escape from the current cluster which may cause handover. At the same time, to maintain effective communication within the cluster, the maximum distance between CM and CH should be less than the coverage of the DSRC, i.e., $\max D\left(V_{CH},V_{CM}\right)<range\left(DSRC\right)$. If $S_{ta}$ remains a very small value, the time duration τ can represent the lifetime of the cluster. The lifetime of cluster can be expressed as

(21) $$T_{lifetime}^{c}=Duration\left(\tau \right)\;\;s.t.\;\;S_{ta}<\delta .$$

The CE performs AD mode recognition based on the collected FCD of CAVs. Based on the location information, the CAVs with the same or similar AD mode are formed into a cluster and generate an info table of the cluster. When the number of CMs changes greatly, it will cause the average relative distance to change greatly, and the lifetime of the cluster may decrease. Therefore, the smaller the $S_{ta}$, the more stable the cluster.

### 5.2. Communication Performance

The proposed architecture contains multiple clusters, the CH set can be expressed as $CH=\left\{CH_{1},CH_{2},\cdots ,CH_{L}\right\}$, and the CM set of each cluster can be expressed as $CM=\left\{CM_{1},CM_{2},\cdots ,CM_{K}\right\}$. In the analysis of reliable traffic, it is necessary to consider two communication models, within the cluster and outside the cluster. The sum of the reliable traffic of the CAVs is the reliable throughput of the fog cells. Therefore, the V2V reliable traffic between the CM and CH in the cluster can be expressed as

(22) $$C_{i\_v}^{V2V}=\eta_{0}W_{0}R\left(t\right)\;i\_v\in C,$$

(23) $$R\left(t\right)=\log_{2}\left(1+L_{v_{i\_v}}\left(t\right)/S_{ta}\right),$$

where $\eta_{0}$ is the spectrum efficiency, $W_{0}$ is the CM bandwidth, and R(t) represents the reliable transmission index.

We assume that the channel quality index ρ(t) is constant within a time period *T*, it can be detected by the vehicle sensing unit. Through the above CH selection mechanism, the CH can stably and reliably collect vehicle data and upload it to the base station. The V2I reliable traffic between the CH and the infrastructure can be expressed as

(24) $$C_{ch}^{V2I}=\rho \left(t\right)\eta_{l}W_{ch},$$

where $\eta_{l}$ is the spectrum efficiency and $W_{ch}$ is the bandwidth allocated to CH for V2I communication.

Thus, the reliable throughput of the proposed architecture can be expressed as

(25) $$C_{reliable}^{L}=C_{i\_v}^{V2V}+C_{ch}^{V2I}\;i\_v\in V\;ch\in C,$$

substituting Equations (9)–(11) into the Equation (12) can obtain

(26) $$C_{reliable}^{L}=\left(\eta_{0}W_{0}\log_{2}\left(1+L_{v_{i\_v}}\left(t\right)/S_{ta}\right)+\rho \left(t\right)\eta_{l}W_{ch}\right)\;v_{i\_v}\in V\;ch\in CH.$$

It can be seen that in order to increase reliable throughput, the stability of the cluster needs to be improved. The improvement of cluster stability can effectively avoid the migration of CAVs between different clusters, thus, the handover and interruption can be avoided and improve cluster lifetime. Based on the proposed AD mode clustering and CH selection mechanism, CAVs can be automatically clustered, which can effectively enhance the cluster stability and reduce handover.

## 6. Simulation Results and Discussions

In this section, simulations and discussions are carried out. We mainly discuss the accuracy of the AD pattern recognition and the reliability of cluster communication to evaluate our proposed architecture and clustering mechanism. The CAVs were equipped with communication equipment and had large numbers of onboard sensors as data sources, such as acceleration, speed, brakes, LiDAR, and other sensors [43].

### 6.1. Training and Test Dataset

**Figure 3:**
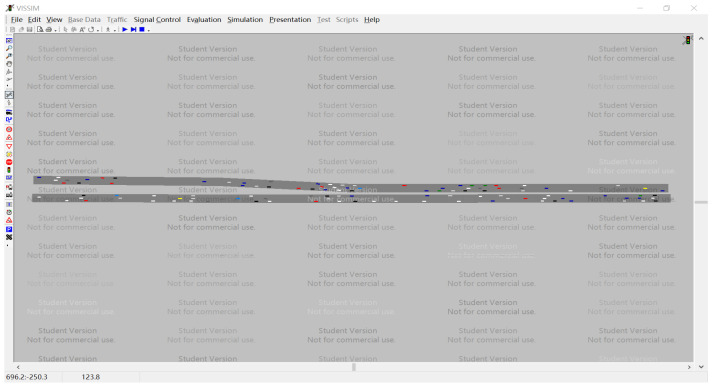
One snapshot of the simulation environment.

In order to verify the rationality of the proposed three AD modes, the traffic simulation software VISSIM was used to collect the sample data. Three experimental vehicle models were built in VISSIM, corresponding to the three driving modes in Section 3.2. By setting the operating parameters of the experimental vehicle, including the desired speed distribution, maximum acceleration function, and desired acceleration function, we established the distribution of three vehicle categories corresponding to the three driving modes. Finally, the data obtained were analyzed to obtain the driving characteristics of the vehicles in the different modes. The settings of the simulation parameters in VISSIM are shown in Table 3. A snapshot of the simulation running environment is shown in Figure 3.

Through statistical analysis of the dynamic driving data collected during the VISSIM simulation period, the distribution of these data was obtained. These data belonged to a truncated Gaussian distribution. The mean and variance were obtained by preprocessing. For simulation convenience, corresponding feature vector matrices were simulated in MATLAB according to distribution features. A portion of the normalized training samples utilized for GANN learning optimization are listed in Table 4. In reality, FCD can be transmitted to the CE via CIoVs and processed in CE, or preprocessed locally.

### 6.2. Performance Analysis of AD Pattern Recognition

The proposed algorithm was trained and validated using the dataset collected by the above method and was compared with the NN without genetic optimization, the deep neural network (DNN) with four layers, and the support vector machine (SVM). The settings of the simulation parameters of the GANN optimization algorithm model are shown in Table 5, which contains the GA part and the NN part. The number of generations and populations must be integers and the values of parameter regarding the probability were decimals between 0 and 1.

**Figure 4:**
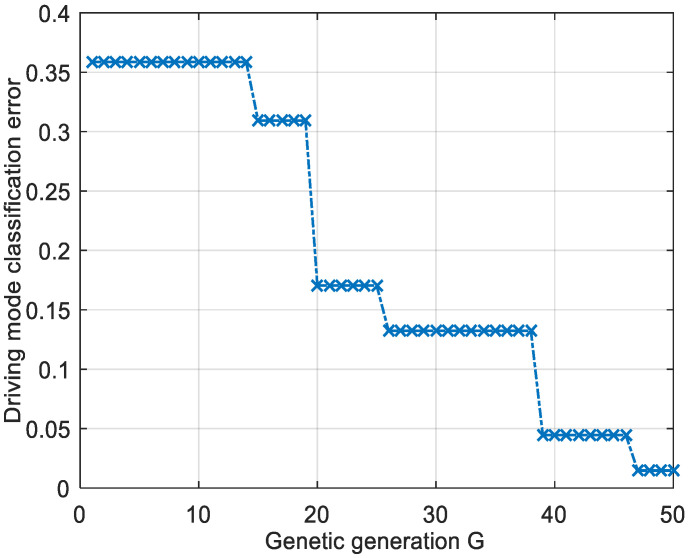
GANN error evolution curve for AD pattern recognition.

Figure 4 shows the evolution of the GANN test errors, which was an important indicator of the generalization ability and accuracy of the proposed GANN pattern recognition algorithm. The test error of the test sample was an important indicator of the generalization ability of the GANN. It can be seen from the figure that the error decreased with the increase in genetic generation. The smaller the test error, the stronger the generalization ability of the network for new samples. The error decreased from above 0.35 to below 0.02 and the minimum test error was 0.014. The minimum value of 0.014 was obtained at 47 generations.

**Figure 5:**
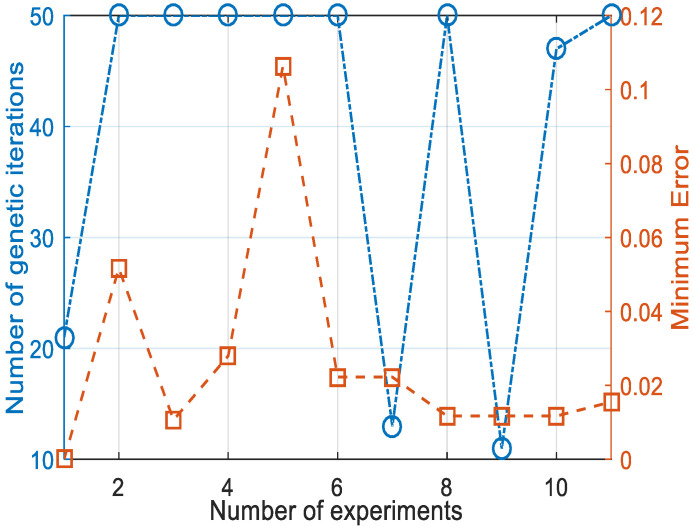
GANN experiment and minimum error.

**Figure 6:**
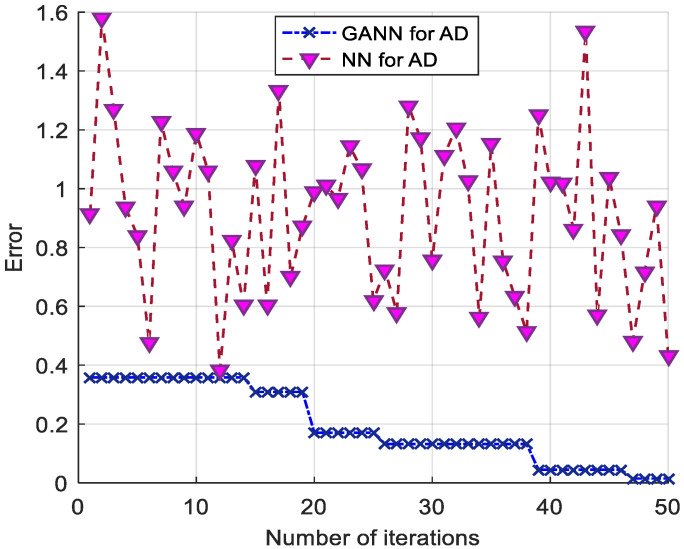
GANN and NN model error iterative evolution.

The number of experiments took into account the balance between cost and effectiveness to verify the reasonableness of the maximum number of genetic generations. It can be seen from Figure 5 that after 50 iterations for each experiment, a low minimum error was achieved. High recognition accuracy was already achieved under this error condition. Therefore, the maximum number of iterations was chosen to be 50 for the tradeoff between efficiency and accuracy. Figure 6 shows the variation curves of the AD pattern recognition errors of GANN and NN. Since the initialization weights and thresholds of each NN were random, the errors converged to different local optimal solutions, and the pattern recognition error fluctuated each time. The GANN for the AD pattern recognition algorithm, which optimized the training error of the NN and continuously reduced the recognition error with genetic iteration, obtained more accurate recognition.

**Figure 7:**
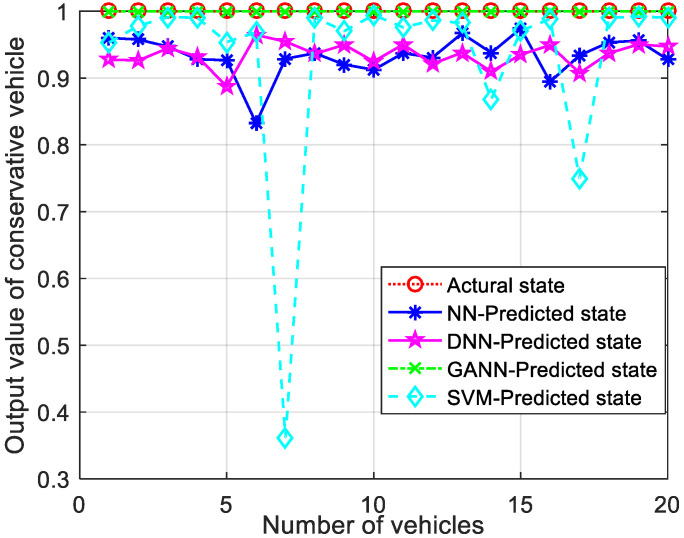
Comparison of output values for conservative CAVs.

Figure 7 shows the comparison of GANN with other ML algorithms for conservative driving pattern recognition. The closer the output value of the pattern recognition algorithm to 1, the higher the recognition accuracy. The output value of the GANN for the AD pattern recognition algorithm was almost equal to 1 and remained stable, while the output values of other algorithms fluctuated significantly. This is because GANN can effectively optimize the initial weights and threshold vectors to obtain the global optimal solution, thus reducing the recognition error. Therefore, the GANN algorithm had higher recognition accuracy and stability than the other algorithms.

**Figure 8:**
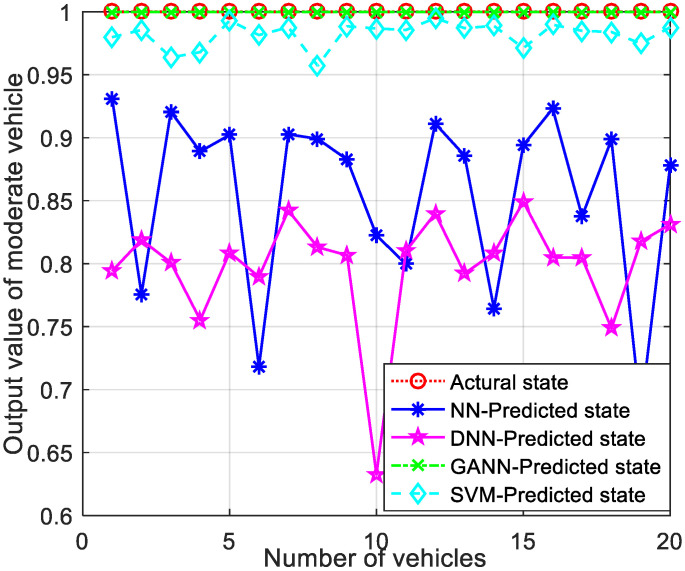
Comparison of output values for moderate CAVs.

**Figure 9:**
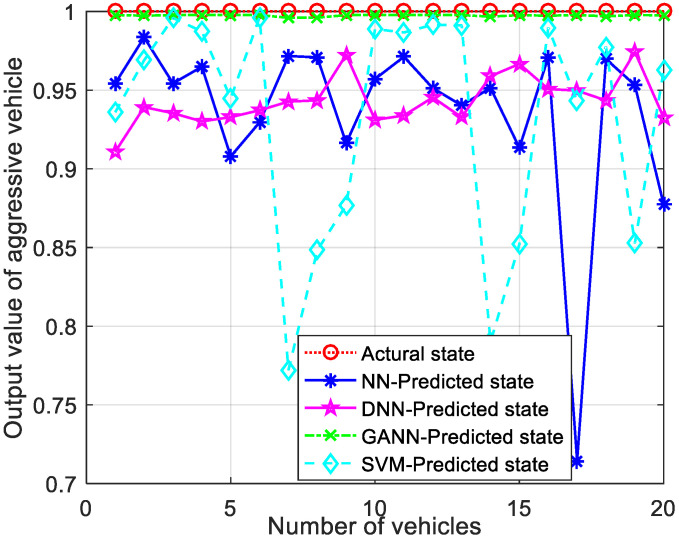
Comparison of output values for aggressive CAVs.

Similarly, to verify the generality of the model, the output comparison simulations for the other two drive models in real life are shown in Figure 8 and Figure 9. The NN and DNN models had a large and dramatic fluctuation between the predicted and actual values. The SVM performed very erratically, due to the jitter of the vehicle driving pattern. However, the predicted values of the GANN model were very close to the actual values, and the fluctuations of the output values were very small. The proposed GANN for AD pattern recognition algorithm had better recognition stability and generalization and was able to perform pattern recognition with high accuracy, which led to more stable clustering.

The test error and training error of different algorithms are shown in Table 6. The proposed GANN for AD pattern recognition algorithm had the smallest error, which could effectively avoid the wrong decisions caused by misjudgments and maintain the stability of the AD clustering system.

### 6.3. The Effectiveness of the Proposed Clustering Mechanism

Effective driving pattern recognition and classification to form clusters were the basic components of our proposed architecture, which are very important for reliable communication. To verify the effectiveness of the proposed clustering mechanism based on the AD mode (ADMode-cluster), we adopted six schemes for comparison as follows:

1. The non-cluster scheme: The scheme was not clustered, and each vehicle on the road only communicated with the new base station through the allocated 5G-V2I technology [25];
2. The section-cluster scheme: The scheme divided the road into sections of equal length, and vehicles on the same road section formed a cluster [26]. Then the V2V communication within-cluster and V2I between CH and gNB were performed, respectively;
3. The ADMode-cluster scheme: The CE used the proposed pattern recognition algorithm to gather vehicles with the same driving pattern into a cluster. On the basis of the proposed clustering mechanism, a heterogeneous hierarchical network architecture was used to carry out multimode communication to complete AD services;
4. The Mixmode-cluster scheme: At the same time, the vehicles in the cluster had different driving modes, i.e., the CAVs in the cluster had three driving modes;
5. The ADMix-cluster scheme: CAVs with different driving modes on the same road at the same time were divided into different subclusters based on the driving modes, i.e., the driving modes in each cluster were the same;
6. The Kmeans-cluster scheme: It performed an iterative unsupervised clustering algorithm based on the position of the CAVs, thus clustering the vehicles into different clusters [46].

The communication simulation was performed considering a two-parallel three-lane (2×3 lanes) urban road. The total bandwidth was 300 MHz, of which 200 MHz was utilized for 5G V2I communication, and the remaining 100 Mhz was exploited for DSRC V2V communication. The spectral efficiency of the DSRC was defined as 10 bps/Hz, the spectral efficiency of the 5G V2I was 15 bps/Hz, and the communication range of the DSRC was 200 m. The other simulation parameters are shown in Table 5.

**Figure 10:**
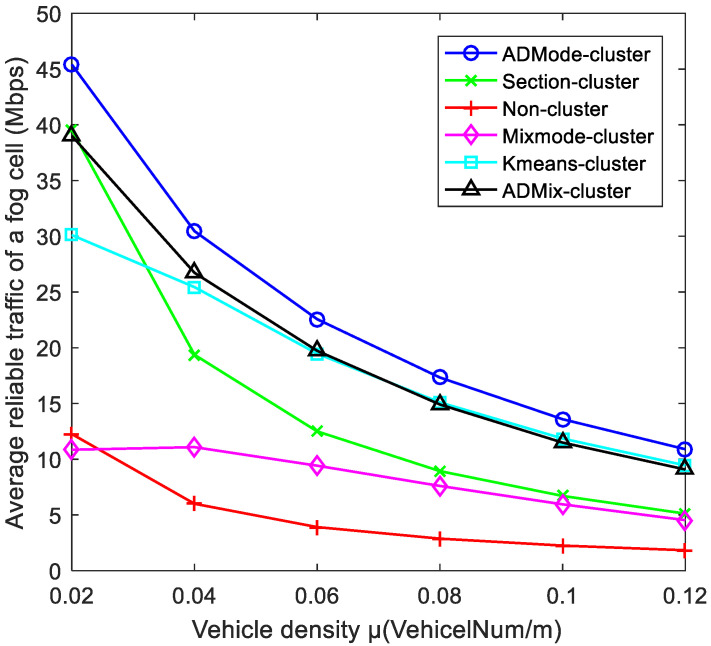
Comparison of average reliable traffic under different vehicle densities.

Figure 10 shows the comparison of the average reliable traffic for the six schemes under different vehicle density conditions. The proposed scheme based on the ADMode-cluster was better than the other five schemes. This is due to the fact that the proposed scheme selects CAVs with the best channel quality and stability as CH. In addition, clusters with the same or similar AD mode are able to maintain stable driving, avoid frequent handover, and interruptions of vehicle communication. Since the ADMix-cluster scheme was divided into different subclusters on the same road, it had better performance in terms of stability. However, too many clusters can cause coverage space overlap and reduce channel utilization. Therefore, the performance was slightly lower than that of the ADMode-cluster scheme. The Kmeans-cluster scheme achieved good results because it performed effective clustering based on location. The non-cluster scheme had the lowest reliable traffic due to the very unstable network topology and only 5G V2I communication mode. The Mixmode-cluster scheme decreased its reliable traffic due to the frequent migration and switching of CAVs with different driving modes within the cluster. As the vehicle density increased, the performance of the Section-cluster was higher than that of the non-cluster scheme but lower than that of the Kmeans-cluster scheme. This is mainly due to the fact that although the section-cluster scheme can use DSRC bandwidth by clustering, this fixed section division scheme cannot perform effective clustering to solve the problem of random movement.

**Figure 11:**
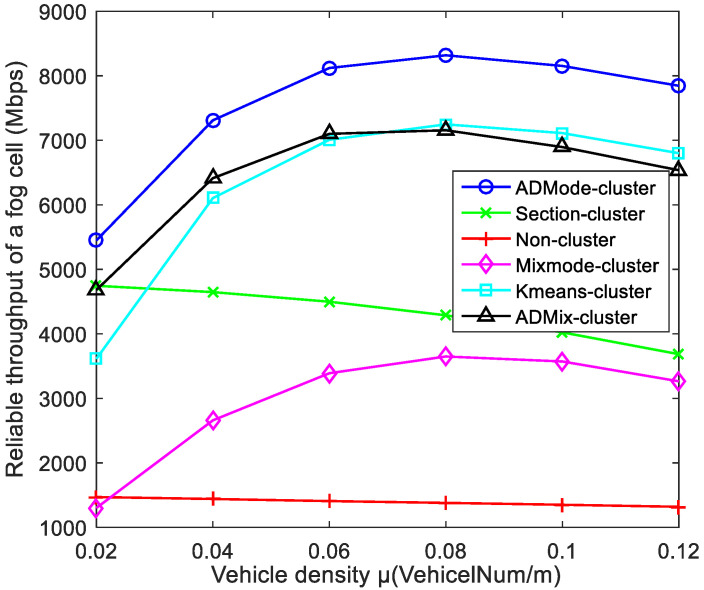
Comparison of reliable throughput under different vehicle densities.

Figure 11 shows the reliable throughputs of the six schemes with different vehicle densities. The throughput of the proposed ADMode-cluster scheme was significantly higher than the other schemes. This is due to the consistent driving mode of the CAVs in the cluster, thus avoiding the frequent replacement of CAVs. Similarly, the ADMix-cluster scheme also achieved high reliable throughput. The section-cluster scheme is a fixed clustering method that divided CAVs into different clusters based on fixed road sections. In this way, the CAVs in the cluster were likely to move out and replace frequently, which would lead to frequent handovers. This phenomenon becomes more pronounced when the vehicle density increases, which will lead to a decrease in reliable throughput. As vehicle density increases, the reliable throughput tends to increase and then decrease. This is mainly because the increase of cluster members results in better spectrum gain but above a certain level will cause an increase in collision probability and overload, thus affecting performance. The non-cluster scheme had the smallest reliable throughput, and the variation with vehicle density was also small since no clustering communication was performed.

**Figure 12:**
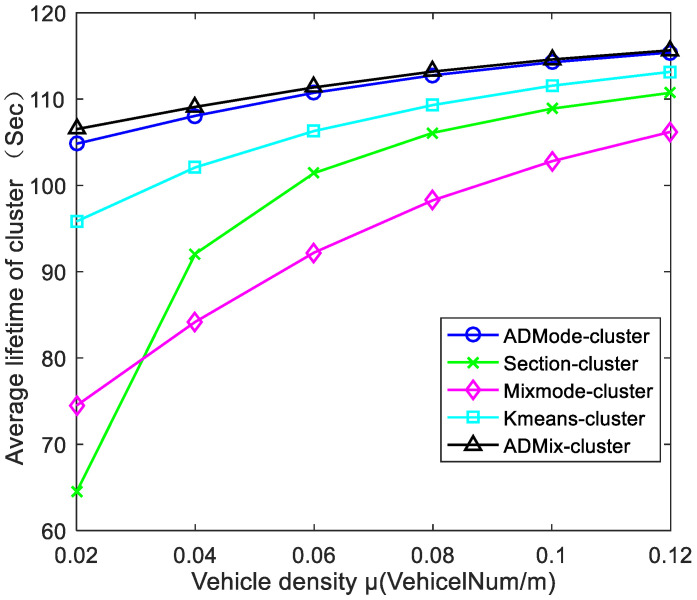
Comparison of average lifetime of cluster under different vehicle densities.

Figure 12 shows the average lifetime of different schemes. The ADMode-cluster scheme and ADMix-cluster scheme had the highest average lifetime of clusters. The reason is that the clusters can be maintained for a long time as the CAVs move in the same driving mode and the driving stability is higher than the hybrid model. This means that CAVs can maintain connections within a cluster for a long time while avoiding frequent handovers. The Kmeans-cluster scheme also showed a good cluster lifetime, since clusters can be kept relatively stable by continuous iterative clustering. Figure 12 shows that the average lifetime of clusters for all schemes tended to increase as the CAVs density increased. In addition, the Mixmode-cluster scheme and Section-cluster scheme were greatly affected by vehicle density. This is because when the vehicle density is higher, there will be more members in the cluster. Therefore, the CAVs are less likely to switch their AD mode, because it becomes difficult to change lanes.

## 7. Conclusions and Future Work

In this paper, we proposed an intelligent clustering mechanism in heterogeneous CIoVs which orchestrated the CAVs into different clusters based on AD mode to enhance the reliability of vehicular communication. In the proposed heterogeneous clustered CIoVs architecture, multiple communication technologies were exploited to improve connectivity and coverage area. We established an AD mode model and proposed a GANN-based AD pattern recognition algorithm to improve the accuracy of the clustering mechanism. Simulation results showed that our approach outperformed the state-of-the-art methods in terms of pattern recognition accuracy, reliable throughput, and cluster lifetime. In future work, we will investigate the blockchain-enabled CIoVs to establish a secure and trustworthy intelligence sharing and incentive platform to facilitate the collaboration of CAVs.

## Figures and Tables

**Table 1:** Comparative study of our work with existing works.

Subject	Ref.	Use Case	Purpose	Contributions
Network architecture	[19]	Vehicular network	Guaranteeing timely data processing or service access	Proposing distributed fog computing for scheduling delay-sensitive applications
	[20]	Vehicular communication	Minimizing transmission costs and fully utilizing communication resources	Proposing a deep Q -learning approach to optimize transmission scheduling
	[21]	Vehicular communication	Improving reliability performance of heterogeneous vehicular communications	Proposing a nearest-first relaying system in the coexistence situation
	[22]	IoV	Evaluating the IoV performance of the scalability and reliability	Modeling and analyzing the performance of V2V and hybrid V2V/V2I networks
	[23]	Vehicular network	Meeting the communication require- ments of the ITS	Proposing a heterogeneous vehicular network with V2I and V2V communications
Network clustering and switching	[24]	Data collection	Guaranteeing QoS and reduce cellular bandwidth cost	Proposing a one-hop clustering and data delivery optimization approach
	[25]	Vehicular communication	Providing high reliability, low latency, and wide area coverage	Proposing a self-adaptive clustering method to adjust clusters
	[26]	Vehicular networks	Analyzing the cluster-based hete- rogeneous vehicular networks	Developing a heterogeneous vehicular networks framework
	[27]	Cognitive radio	Increasing network scalability in a distributed cognitive radio network	Proposing a novel reinforcement learning based trust model to adjust cluster size
	[28]	Network selection	Improving quality of service of vehicles	Developing a network recommendation system through big data analysis
	[29]	Vehicular networks	Making load balancing among hetero- geneous base stations	Proposing an online reinforcement learn- ing approach for network load balancing
Vehicle clustering and driving behavior	[30]	Autonomous vehicle	Clustering a wide range of driving encounter scenarios	Proposing a generic unsupervised learing framework
	[31]	IoV	Dealing with large scale network-based trajectory data	Designing a dynamic network representa- tion learning based clustering method
	[32]	Transportation management	Extracting travel patterns from large- scaled vehicle trajectories	Developing a calculation process for clustering vehicle trajectory data
	[33]	ITS	Achieving traffic flow control in Social IoV	Presenting a social vehicle route sele- ction algorithm to select optimal route
	[34]	Autonomous driving	Designing safe, smart, and personalized autonomous driving systems	Proposing a driving model to emulate a human decision making mechanism
	[35]	Driving behavior	Supporting automated driving systems and human-like decision making	Proposing a unified model of driving behavior in different scenarios
	[36]	Driving behavior	Identifying abnormal driving behaviors to prevent traffic accidents	Proposing different abnormal driving behavior recognition algorithms
	[37]	ITS	Monitoring driver behavior to reduce traffic accident risk	Presenting a novel deep learning method for analyzing driver behavior
	Our work	Autonomous driving	Providing stable communication connections between vehicles	Proposing an intelligent clustering mech- anism based on driving patterns

**Table 2:** Neural network variables and parameters.

Network Layer	Input Layer	Hidden Layer	Output Layer
Neuron Number	$n_{1}=6$	$n_{2}=42$	$n_{3}=3$
Transfer Function	*purelin*	*tansig*	*softmax*
Variables	$n_{1}$	$n_{2}$	$n_{3}$
Input	$x_{i}$	$y_{j}$	$z_{k}$
Output		$O_{j}$	$Z_{k}$
Weights	$w_{ji}$	$w_{kj}$	
Thresholds		$\theta_{j}$	$\theta_{k}$

**Table 3:** Simulation configuration in VISSIM.

Simulation Parameter	Value
Road length	1000 m
Number of lanes	3
Road type	Urban motorway
Car following model	Wiedemann 99
Traffic flow	3000
Vehicle mode	3
Simulation period	360 s

**Table 4:** Normalized feature factors of examples.

Num	1	2	3	4	5	6	7	8
Fac								
***A***	0.1280	0.0815	0.1104	0.6400	0.5059	0.4983	0.9466	0.8753
***B***	0.1641	0.6104	0.4600	0.3347	0.3403	0.3024	0.6179	0.4628
***C***	0.2485	0.0394	0.0001	0.4362	0.3578	0.1700	0.5369	0.8277
***D***	0.5683	0.1800	0.6096	0.5493	0.7245	0.4920	0.4391	0.7612
***E***	0.0580	0.0692	0.0350	0.4408	0.4151	0.4485	0.9228	0.9372
***F***	0.2076	0.0963	0.1011	0.4100	0.3530	0.3778	0.8139	0.8721
Cons	1	1	1	0	0	0	0	0
Mod	0	0	0	1	1	1	0	0
Aggr	0	0	0	0	0	0	1	1

**Table 5:** Simulation parameters.

Simulation Parameter	Value
Population size M	40
Genetic generation G	50
Cross probability px	0.7
Mutation probability pm	0.01
Generation gap GGAP	0.95
Training goal Tg	0.01
Learning rate Lr	0.1
DSRC Channel bandwidth [44]	10 MHz
5G V2I Channel bandwidth [45]	20 MHz
Total bandwidth	300 MHz

**Table 6:** Pattern recognition errors.

ML	Test Error	Training Error
NN	1.0568	1.7353
GANN	0.014711	0.023431
DNN	1.1882	1.9773
SVM	0.88613	0.17789

## Data Availability

The data used to support the findings of this study are available from the corresponding author upon request.

## Abbreviations

CIoVs	Cognitive Internet of Vehicles
ITS	Intelligent Transportation System
AD	Autonomous Driving
CAVs	Connected Autonomous Vehicles
NLOS	Non Line of Sight
IoV	Internet of Vehicles
CE	Cognitive Engine
AI	Artificial Intelligence
CH	Cluster Head
DSRC	Dedicated Short Range Communication
V2I	Vehicle to Infrastructure
GA	Genetic Algorithm
NN	Neural Networks
SDN	Software Defined Network
RSU	Roadside Unit
CR	Cognitive Radio
V2V	Vehicle to Vehicle
CM	Cluster Member
gNB	5G Base Station
CS	Cloud Server
FCD	Floating Car Data
ML	Machine Learning
ECU	Electronic Control Unit
DNN	Deep Neural Network
SVM	Support Vector Machine
